# Inhibition of Inducible Heat Shock Protein-70 (Hsp72) Enhances Bortezomib-Induced Cell Death in Human Bladder Cancer Cells

**DOI:** 10.1371/journal.pone.0069509

**Published:** 2013-07-18

**Authors:** Wei Qi, Matthew C. White, Woonyoung Choi, Charles Guo, Colin Dinney, David J. McConkey, Arlene Siefker-Radtke

**Affiliations:** 1 Department of Urology, U. T. M. D. Anderson Cancer Center Houston, Texas, United States of America; 2 Department of Cancer Biology, U. T. M. D. Anderson Cancer Center Houston, Texas, United States of America; 3 Department of Pathology, U. T. M. D. Anderson Cancer Center Houston, Texas, United States of America; 4 Department of Genitourinary Medical Oncology, U. T. M. D. Anderson Cancer Center Houston, Texas, United States of America; 5 The Graduate School of Biomedical Sciences, University of Texas Health Sciences Center, Houston, Texas, United States of America; University of Wisconsin School of Medicine and Public Health, United States of America

## Abstract

The proteasome inhibitor bortezomib (Velcade) is a promising new agent for bladder cancer therapy, but inducible cytoprotective mechanisms may limit its potential efficacy. We used whole genome mRNA expression profiling to study the effects of bortezomib on stress-induced gene expression in a panel of human bladder cancer cell lines. Bortezomib induced strong upregulation of the inducible HSP70 isoforms HSPA1A and HSPA1B isoforms of Hsp72 in 253J B-V and SW780 (HSPA1A^high^) cells, but only induced the HSPA1B isoform in UM-UC10 and UM-UC13 (HSPA1A^low^) cells. Bortezomib stimulated the binding of heat shock factor-1 (HSF1) to the HSPA1A promoter in 253JB-V but not in UM-UC13 cells. Methylation-specific PCR revealed that the HSPA1A promoter was methylated in the HSPA1A^low^ cell lines (UM-UC10 and UM-UC13), and exposure to the chromatin demethylating agent 5-aza-2′-deoxycytidine restored HSPA1A expression. Overexpression of Hsp72 promoted bortezomib resistance in the UM-UC10 and UM-UC13 cells, whereas transient knockdown of HSPA1B further sensitized these cells to bortezomib, and exposure to the chemical HSF1 inhibitor KNK-437 promoted bortezomib sensitivity in the 253J B-V cells. Finally, shRNA-mediated stable knockdown of Hsp72 in 253J B–V promoted sensitivity to bortezomib *in vitro* and in tumor xenografts *in vivo*. Together, our results provide proof-of-concept for using Hsp72 inhibitors to promote bortezomib sensitivity in bladder cancers and suggest that selective targeting of HSPA1B could produce synthetic lethality in tumors that display HSPA1A promoter methylation.

## Introduction

Recent studies have established that increased protein synthesis (translation) is crucial for neoplastic transformation. As a consequence of this increase, cancer cells appear to be particularly vulnerable to agents inhibiting the elimination of aggregated or misfolded proteins produced as a normal byproduct of protein synthesis. The proteasome plays a central role in the clearance of damaged proteins, and proteasome inhibitors induce tumor cell death in large part via protein aggregation and proteotoxicity. However, cytoprotective mechanisms are upregulated by proteasome inhibition, limiting the impact on cancer cell death [1], [2], [3]. Therefore, it is possible that tumors that possess defect(s) in these cytoprotective mechanisms will be especially sensitive to proteasome inhibitors. If the relevant cytoprotective mechanisms can be identified, it may be possible to identify these tumors prospectively. Alternatively, it may be possible to develop therapeutic approaches that disrupt these cytoprotective mechanisms thereby promoting proteasome inhibitor sensitivity in tumors that would otherwise be resistant to this class of drugs.

Heat shock also induces protein aggregation and proteotoxicity with heat shock proteins (HSPs) promoting heat tolerance by preventing inappropriate stress-induced protein aggregation, assisting in the proper refolding of denatured proteins, and, if necessary, promoting their degradation [4], [5], [6]. Members of the Hsp70 family are among the most highly conserved proteins in evolution and play critical roles in these processes [7]. The major stress-inducible member of the Hsp70 chaperone family is referred to as Hsp72 and is encoded by two genes, HSPA1A and HSPA1B, which produce Hsp72 isoforms that share all but two amino acids and are thought to be functionally redundant [8]. Hsp72 expression is controlled via rapid activation of heat shock factor-1 (HSF1), a transcription factor that binds to several specific response elements located within the Hsp72 isoform promoters and the promoters of other heat-induced cytoprotective chaperones [9]. Hsp72 is highly homologous to the 78 kDa glucose-regulated protein (HSPA5, GRP78/BiP) that plays a central role in coordinating the activation of the unfolded protein response (UPR) and is also induced by proteasome inhibitors [10]. Hsp72 is constitutively expressed at high levels in malignant tumors of various origins [11], [12], promoting cancer cell survival [13], [14]. Importantly, Hsp72 is also robustly induced by proteasome inhibitors [15], [16].

In a previous study we reported that approximately half of human bladder cancer cells are resistant to the cytotoxic effects of bortezomib *in vitro* [17]. Here, we used gene expression profiling to examine cytoprotective mechanisms that contribute to bortezomib resistance, and found that Hsp72 is one of the most robustly induced genes at the mRNA level following bortezomib exposure. However, we found that Hsp72 expression is isoform-specific in a subset of bladder cancer cells (UM-UC10 and UM-UC13) as a result of promoter methylation of the HSPA1A isoform. These cells display increased expression of the HSPA1B isoform such that basal and bortezomib-induced Hsp72 protein levels are similar to those found in cell lines that express both A1A and A1B isoforms (253JB-V and SW780). We also report that suppression of Hsp72 induction enhanced bortezomib-induced cell death in both 235JB-V and UM-UC10, and overexpression of Hsp72 protected UM-UC10 and UM-UC13 cells from bortezomib-induced cytotoxicity. Overall, regardless of which isoform generates Hsp72 protein expression, our data show that suppression of Hsp72 induction enhances bortezomib sensitivity, and support the further development of HSF1 and Hsp72 inhibitors to increase bortezomib sensitivity in bladder cancers.

## Materials and Methods

### Cell lines and Reagents

Bladder cancer cell lines were obtained from the MD Anderson Bladder Cancer SPORE Cell Line Repository and maintained in MEM supplemented with 10% fetal bovine serum (FBS) (Omega Scientific, Tarzana, CA). The authenticity of all of the cell lines was confirmed at deposit by DNA fingerprinting, and their identities were routinely confirmed during experimentation in the MD Anderson Characterized Cell Line Core [18]. Cell line 253-JB-V, [19] UMUC-10, [20] and UMUC-13 [20] were isolated from patients with urothelial cancer. SW780 was originally purchased from American Type Culture Collection (ATCC) at Biocompare.com. Bortezomib was purchased from ChemieTek (IN, USA). For *in vitro* experiments, bortezomib was dissolved in DMSO at a stock concentration of 10 mM, sterilized by filtration through a 0.22 µm syringe filter, with aliquots stored at −20°C until use. Prior to use, the stock was diluted in medium to the desired concentrations. For injection of mice, bortezomib was dissolved in saline containing 10 mg/mL mannitol just before treatment.

### Cell Viability Assays

Cells were exposed to bortezomib, collected at the indicated time points by trypsinization, and resuspended in 500 µl PBS. Fifty µl PBS, pH 7.4, containing 100 µg/ml propidium iodide (PI) was added to the resuspended cells, and PI uptake (indicative of cell death) was analyzed immediately by flow cytometry (FACS) on a Cytomics FC 500 with CXP Software (Beckman Coulter, Inc., Fullerton, CA.

For trypan blue exclusion, cells were collected by trypsinization, stained with 0.4% trypan blue (Invitrogen), and cells were counted using a hemocytometer. The experiment was conducted in triplicate.

### Microarray Analyses

Microarray experiments were performed as described previously [21] with minor modifications. RNA was isolated from cells using the TRIzol Reagent (Invitrogen/Life Technologies, Grand Island, NY), followed by cleanup with RNeasy Mini kits (Qiagen, Germantown, MD). RNA was used for the synthesis of biotin-labeled cRNA, which was prepared using the Illumina RNA amplification kit (Ambion/Life Technologies), and then hybridized to Illumina Human-HT12 (Illumina, Inc., Hayward, CA) chips. Washed chips were scanned with BeadStation 500x (Illumina) and the signal intensities quantified with BeadStudio (Illumina). The heatmap was made using Cluster 3.0 and Java Treeview from the Eisen lab (http://www.eisenlab.org/eisen/). The microarray dataset can be found in Gene Expression Omnibus, accession number GSE46132.

### mRNA Extraction, Reverse Transcription and Quantitative Real-time PCR

mRNA extraction and reverse transcription were performed as described previously [22]. RNA was isolated from cells using the TRIzol Reagent (Invitrogen), and cDNA synthesis was performed using SuperScript III First-Strand Synthesis System for RT–PCR (Invitrogen). Real-time PCR for HSPA1A, HSPA8, HSPB1, DNAJB1, and glyceraldehyde-3-phosphate dehydrogenase (GAPDH) was performed using a StepOne real-time PCR system (Applied Biosystems/Life Technologies). The TaqMan primer sets for HSPA1A (Hs00359163_s1), HSPA1B (Hs00271244_s1), pan-HSPA1A & HSPA1B (Hs00271229_s1), HSPA8 (Hs03045200_g1), HSPB1 (Hs03044127_g1), DNAJB1 (Hs00428680_m1), and for GAPDH (4333764F) were purchased from Applied Biosystems. The amplification protocol consisted of one cycle at 50°C for 2 min, one cycle at 95°C for 10 min, followed by 40 cycles at 95°C for 15 s and 60°C for 60 s, and transcript levels were quantified using the comparative C_T_ method. The resulting data were analyzed with StepOne software and expressed as the mean of ratios (relative expression to control) ± SE, and GAPDH served as the internal loading control.

### Treatment of Cells with 5-aza-2′-deoxycytidine (5-AzdC)

Cells were plated at low density (∼5×10^4^ cells/well) in 6-well plates and allowed to attach overnight. Cells were then exposed to 5 µM 5-AzdC dissolved in 50% acetic acid for 5 days. Bortezomib (30 nM) was then added to appropriate wells 6 hours prior to harvesting on day 5, and cells were collected for RNA isolation. 5-AzdC was obtained from Sigma.

### DNA Methylation Analysis

Genomic DNA was isolated using a genomic DNA isolation kit (Qiagen). DNA (1 µg) was converted with sodium bisulfite using the EpiTect Bisulfite Kit (Qiagen) according to the manufacturer’s instructions. The bisulfite-modified DNA was then subjected to methylation-specific PCR (MSP). The primers used for MSP were designed using Methprimer. The primer set for converted methylated DNA was 5′-TGTTTTTTTTATTCGGATTAGTTAAC-3′ (forward) and 5′-CCACCTACTCGCTAAAACTACGTA-3′ (reverse); The primer set for converted unmethylated DNA was 5′- TTTTTTTTATTTGGATTAGTTAATGT -3′ (forward) and 5′- CCCACCTACTCACTAAAACTACATA -3′ (reverse). The PCR protocol included an initial incubation at 95°C for 10 min, followed by 35 cycles of 95°C for 30 s, 49°C for 30 s and 72°C for 40 s, followed by one cycle of 72°C for 10 min. MSP PCR products were separated on 2% agarose gels and visualized by ethidium bromide staining. Fully methylated control DNA and unmethylated control DNA were used as controls.

### Immunoblotting

Cells were harvested by trypsinization and lysed in buffer containing 20 mM Tris-HCl (pH 7.5), 150 mM NaCl, 1 mM Na_2_EDTA, 1 mM EGTA, 1% Triton, 2.5 mM sodium pyrophosphate, 1 mM beta-glycerophosphate, 1 mM Na_3_VO4, 1 µg/ml leupeptin and 1 mM PMSF. Whole-cell extracts (20 µg total protein) were subjected to sodium dodecyl sulfate-10% polyacrylamide gel electrophoresis (SDS-PAGE) and transferred to nitrocellulose membranes (Bio-Rad, Hercules, CA). Membranes were probed first with either a monoclonal antibody specific for the Hsp72 (SPA-810, Stressgen/Enzo Life Sciences, Farmingdale, NY), HSF1 (SPA-901, Stressgen/Enzo) or human beta-actin (Sigma, St. Louis, Mo.), and then with appropriate horseradish peroxidase-conjugated second antibodies (Santa Cruz Biotechnology, Dallas, TX). Immunodetection was performed using ECL (Amersham, Piscataway, N.J.) according to manufacturer’s instructions.

### Chromatin Immunoprecipitation

Chromatin Immunoprecipitation (ChIP) was performed with the ChIP-IT™ Express Enzymatic kit, and ChIP-IT™ Control Kit (Active Motif) according to the manufacturer’s protocol. Control and bortezomib-treated 253JB-V and UM-UC13 cells (1.5×10^7^ each) were fixed for 8 minutes at room temperature and sheared by enzymatic digestion for 10 minutes. The sheared chromatin yielded bands between 200–1500 bp as visualized by agarose gel electrophoresis. DNA bound to HSF1 was precipitated with an anti-HSF1 antibody (Stressgen/Enzo, SPA-901). To amplify the HSF1-bound HSPA1A promoter, the precipitated DNA was subjected to real-time PCR using the TaqMan® Gene Expression Master Mix with Custom TaqMan® Gene Expression Assay primers (ABI) corresponding to the HSF1-binding region of the HSPA1A promoter (−10 to −180) [23], [24]. Real-time PCR was performed using ABI StepOne with following conditions: 5 minutes at 50°C; 10 minutes at 95°C; then 40 cycles of 95°C for 15 seconds, 60°C for 60 seconds. The data presented represent results from three separate ChIP experiments and were normalized to reactions performed with 1% of input. End-point PCR reactions were also performed as described previously [25] to confirm the real-time PCR results. Normal IgG antibody was used as a control.

### Molecular Modulation of Hsp72 Expression

The lentiviral pLKO.1-based constructs TRCN0000008762 and TRCN0000008757 specifically targeting the Hsp72 gene were purchased from Open Biosystems, Inc. The empty pLKO.1 vector was used as a control. Recombinant viruses were produced by calcium phosphate transfection of HEK293T cells using standard protocols. At day 2–3 post-culture, 253J-BV cells were incubated with shRNAs and polybrene (6 µg/ml) for 16∼24 hours, and the transduced cells were selected in 1 µg/ml puromycin. For transient silencing of Hsp72 in UM-UC10 cells, cells were incubated in 6 or 24-well plates for 72 hours with either non-targeting (D-001206-14-20), siHSPA1A (L-005168-00), or HSPA1B (L-003501-00) siRNA constructs from Dharmacon/Thermo Scientific, Waltham, MA. Lipofectamine RNAiMAX transfectionreagent (Invitrogen) was used to enhance siRNA delivery according to manufacturer’s instructions. For overexpression of Hsp72, the Precision LentiORF RFP control (OHS5832) and Precision LentiORF individual clone for HSPA1A (OHS5897–100998480) were purchased from Open Biosystems, Inc. Transduced cells were selected in 5 µg/ml blasticidin and FACS sorting of GFP positive cells.

### Lysosomal Integrity Assays

Cells (∼1–2×10^5^) were plated in 6-well plates and allowed to attach overnight. Cells were then exposed to bortezomib for 24 h. Following drug treatments, 100 nM LysoTracker Red DND-99 (Molecular Probes/Life Technologies, Grand Island, NY) was added to cells for 30 minutes prior to harvest. Cells were trypsinized, washed once with PBS, and resuspended in fresh PBS, and fluorescence was measured using a Beckman Coulter FC500 flow cytometer.

### Xenograft Studies

Female athymic nude mice were purchased from National Cancer Institute (NCI-Frederick). The mice were housed and maintained under specific pathogen-free conditions in facilities approved by the American Association for Accreditation of Laboratory Animal Care and in accordance with current regulations and standards of the United States Department of Agriculture, United States Department of Health and Human Services. This study was carried out in strict accordance with the recommendations in the Guide for the Care and Use of Laboratory Animals of the National Institutes of Health. The protocol was approved by the University of Texas M. D. Anderson Cancer Center Animal Care and Use Committee (ACUF Protocol # 11-00-12734). The mice are monitored daily, including weekends and holidays, with euthanasia performed using CO2 in the event of any of the following: tumor greater than 1.5 cm, >20% weight loss, lethargy, inability to obtain food and/or water, labored breathing, hunched posture, abdominal distension equivalent to a pregnant mouse, or incapacitated as a result of tumor growth.

Nude mice (NIH, 6 weeks of age) were inoculated subcutaneously (s.c.) with 2×10^6^ 253J B-V cells transduced with the HSPA1A shRNA construct (253JB-V.KDHsp72) or a non-targeting control construct (253JB-V.NT) (10 mice/group). When tumors became palpable (5∼7 days), the mice were randomly assigned to control or treatment groups. The mice were treated i.v. (via the tail vein) biweekly with 1 mg/kg bortezomib formulated in saline containing 10 mg/mL mannitol in a volume of 100 µl or with 100 µl saline containing 10 mg/mL mannitol as a vehicle control. Caliper measurements of the longest perpendicular tumor diameters were performed twice a week after the start of treatment, and the volumes of tumors were calculated using the formula: W*W*L/2 (where W and L represented transverse diameter, and longest longitudinal). For H&E analysis, tumors were collected from mice 24 hours after the second drug treatment and then fixed in OCT and 10% formalin.

### UCSC Genome Browser

The UCSC Genome Browser [26] was used to identify the presence of a CpG island surrounding the HSPA1A promoter region, Within the Genome Browser, the encyclopedia of DNA elements consortium (ENCODE) database [27] was used to identify the presence of methylation in other cell types. The UCSC Genome Browser is publically available at the following site: http://genome.ucsc.edu/.

### Statistics

Statistics were generated using the Student’s *t* test functions available in GraphPad Prism 5 and Microsoft Excel software. P-values of <0.05 were considered to be significant.

## Results

### Differential Induction of HSPA1A in Bladder Cancer Cells

**Figure 1:**
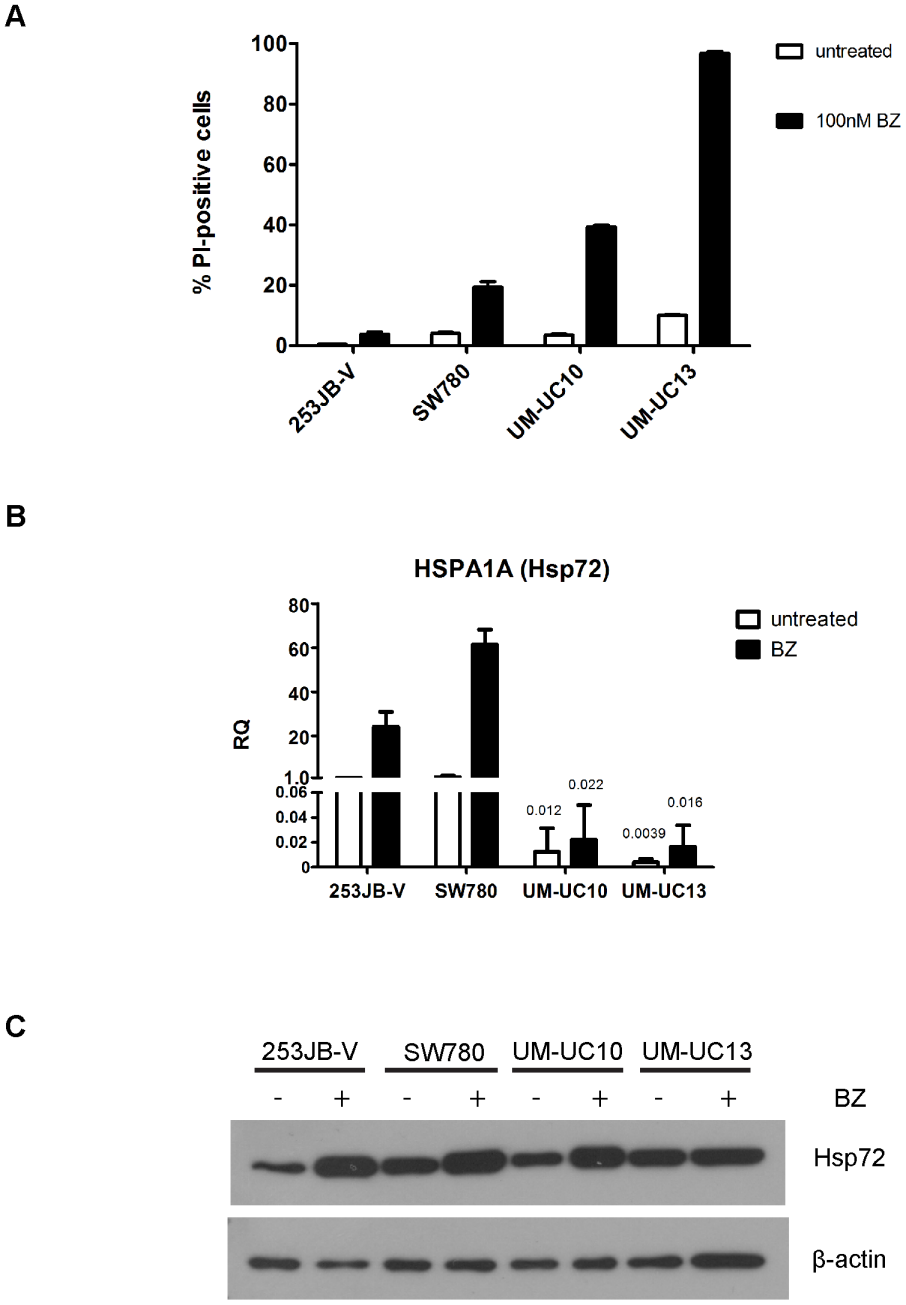
Effects of bortezomib on cell death and Hsp72 mRNA and protein expression in a subset of bladder cancer cells. **A.** Effects of bortezomib on cell death. Bladder cancer cell lines (253JB-V, SW780, UM-UC10, UM-UC13) were incubated with or without 100nM bortezomib for 24 hours and PI/FACS was used to quantify cell death. Mean ± SEM, n = 3. **B.** Effects of bortezomib on the mRNA expression of Hsp72 isoform HSPA1A. Cells were exposed to 30 nM bortezomib for 6 h and HSPA1A was measured by quantitative RT-PCR. RQ = relative quantity (to GAPDH). Values represent mean ± SEM (N≥3) **C.** Effects of bortezomib on Hsp72 protein levels. Cells were incubated for 16–18 h with 30 nM of bortezomib (nM), and Hsp72 levels were measured in whole cell lysates by immunoblotting. Blots are representative of two independent experiments.

We selected four representative human bladder cancer cell lines (253J B-V, SW780, UM-UC10, and UM-UC13) for characterization of the molecular biological mechanisms that determine cellular responsiveness to the proteasome inhibitor bortezomib. We first confirmed that the cell lines were heterogeneous with respect to their sensitivities to bortezomib-induced cell death as determined using propidium iodide staining and FACS analysis (PI-FACS) to measure loss of plasma membrane integrity (Fig. 1A). We then used whole genome mRNA expression profiling (Illumina platform) to identify the gene expression patterns associated with drug sensitivity and/or resistance. Bortezomib induced strong upregulation of mRNA encoding the major inducible isoform of Hsp72 (HSPA1A) in the most bortezomib-resistant cell line (253J B-V) but not in the most drug-sensitive line (UM-UC13) (Fig. S1). We confirmed these results using quantitative real-time RT-PCR, demonstrating that HSPA1A mRNA was strongly induced by bortezomib in 253JB-V and SW780 cells (∼25–60 fold over untreated levels), whereas expression increased only slightly induced (∼2–4 fold over untreated levels) in UM-UC10 and UM-UC13 cells (Fig. 1B). We also noticed very low basal HSPA1A mRNA expression in UM-UC10 and UM-UC13 cells (∼50–250 fold lower than 253JB-V and SW780) and these differences were exacerbated upon bortezomib exposure such that HSPA1A expression levels were ∼1000–3000 fold lower in UM-UC10 and UM-UC13 cells than in 253JB-V and SW780 (Fig. 1B). However, immunoblotting revealed comparable Hsp72 protein levels in all 4 cell lines (Fig. 1C).

### HSPA1B Isoform Compensates for Loss of HSPA1A Expression in UM-UC10 and UM-UC13 Cells

**Figure 2:**
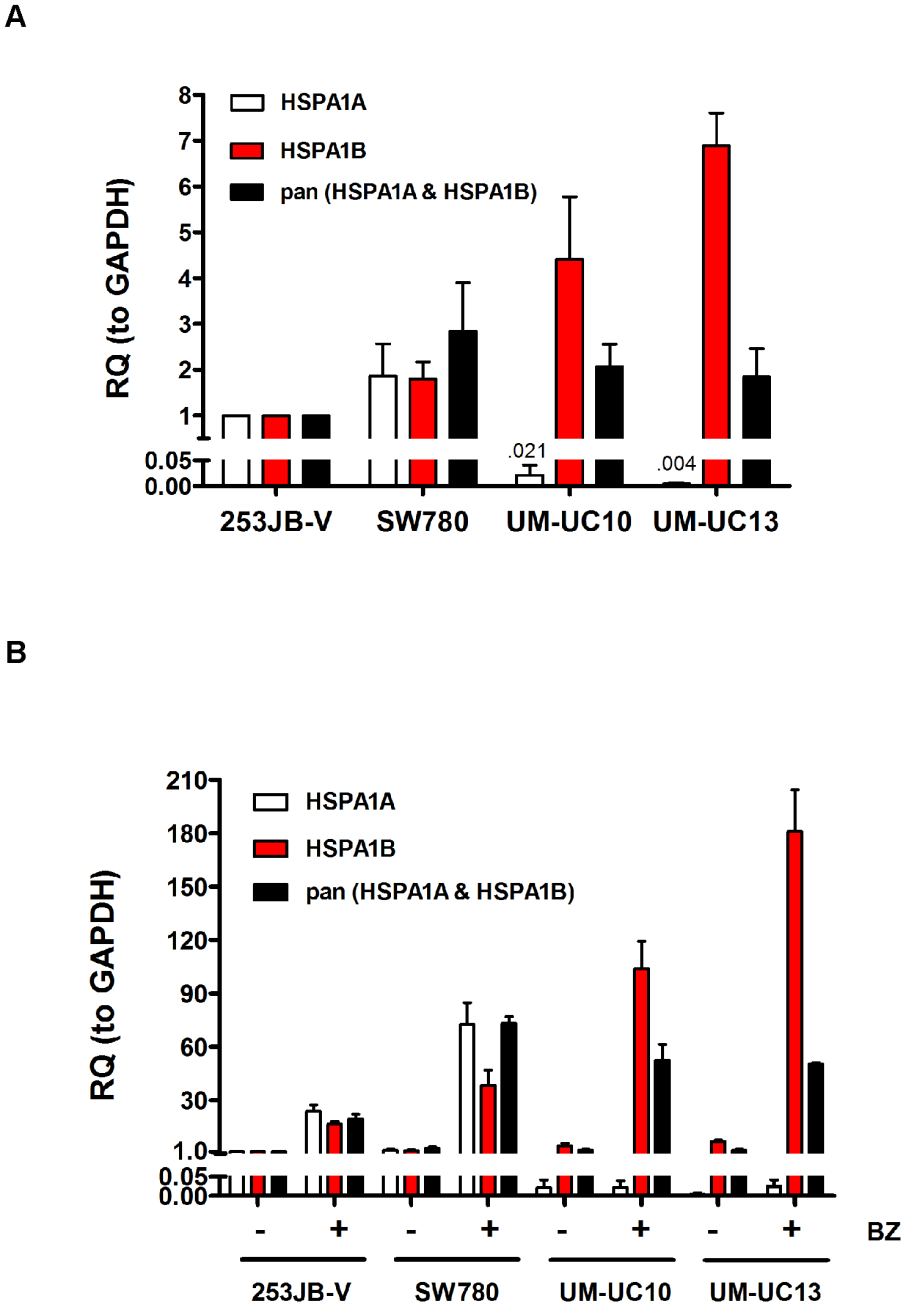
Increased HSPA1B expression compensates for loss of HSPA1A expression in UM-UC10 and UM-UC13 cells. **A.** Basal expression of HSPA1A and HSPA1B isoforms across the four cell lines. **B.** Bortezomib-induced expression of HSPA1A and HSPA1B across the four cell lines. Specific primers for each isoform, as well as a pan-primer that recognized both isoforms, were used to measure expression by quantitative RT-PCR. Values represent mean±SE (n = 2). RQ = relative quantity (to GAPDH).

Hsp72 is encoded by two independent genetic loci (HSPA1A and HSPA1B) that produce highly homologous protein products. We therefore characterized HSPA1B expression in the HSPA1A^low^ cells. We used primers specific for the two isoforms of Hsp72, HSPA1A and HSPA1B, as well as a primer that recognized both (pan) isoforms for comparison. Our data revealed that the HSPA1A^low^ cells (UM-UC10, UM-UC13) had higher expression of the HSPA1B isoform at baseline than did the HSPA1A-high cells (253JB-V, SW780) (Fig. 2A). In addition, HSPA1B expression was more robustly induced following bortezomib exposure in the HSPA1A^low^ cells lines that lacked the A1A isoform (Fig. 2B). Importantly, expression measured by the pan-primer was similar across all four cell lines, corroborating the immunoblotting data (Fig. 1C). These data suggest that increased HSPA1B expression compensated for the lack of HSPA1A and accounted for the Hsp72 protein expression in the UM-UC10 and UM-UC13 cells.

### Lack of HSPA1A Inducibility in UM-UC10 and UM-UC13 Cells is due to Promoter Methylation

**Figure 3:**
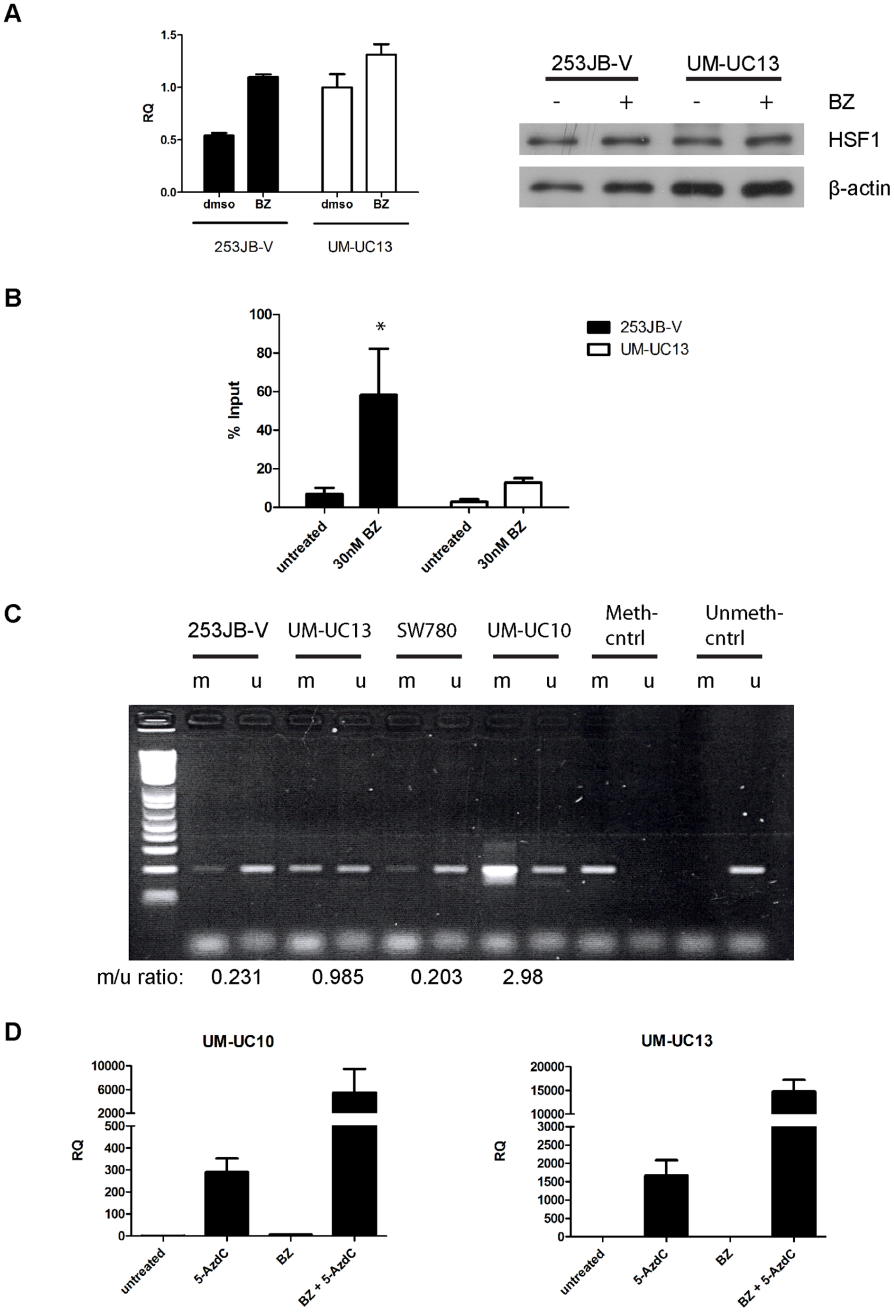
Selective methylation of the HSPA1A promoter suppresses gene expression in UM-UC10 and UM-UC13 cells. **A.** Expression of HSF1. 253J B-V and UM-UC13 bladder cancer cell lines were exposed to bortezomib for 12 h and HSF1 mRNA (left panel) and protein levels (right panel) were measured by quantitative RT-PCR and immunoblotting, respectively. Values represent mean±SE (n = 2); blots are representative of at least two independent experiments. **B.** Chromatin immunoprecipitation (ChIP) analysis of HSF1 binding to the HSPA1A promoter. Note that basal and bortezomib-induced HSF1 binding was greatly reduced in the bortezomib-sensitive UM-UC13 cells. Mean ± SEM, n = 3. *P<0.05 **C.** Selective methylation of the HSPA1A promoter in bortezomib-sensitive human bladder cancer cells. Methylation-specific PCR was used to assess chromatin methylation in drug-sensitive (UM-UC10, UM-UC13) and drug-resistant (253J B-V, SW780) cell lines as described in Materials and Methods. m, methylated; u, unmethylated. m/u ratios were calculated using densitometry. Data are representative of at least two independent experiments. **D.** The DNA methyltransferase inhibitor 5-aza-2′-deoxycytidine restores HSPA1A expression in bortezomib-sensitive cells. Cells were incubated with 5 µM 5-AzdC for 5 days and then incubated with or without 30nM bortezomib for 6 h, and HSPA1A expression was measured by quantitative real-time PCR. Values represent mean ± SEM, n = 3. RQ = relative quantity (to GAPDH).

Heat shock factor-1 (HSF1) activation controls the global heat shock response and stress-induced upregulation of Hsp72 [28]. To test whether HSF1 expression was influencing differences in HSPA1A expression among our cell lines, we measured HSF1 mRNA and protein levels in the 253JB-V (HSPA1A^high^) and UM-UC13 (HSPA1A^low^) cells. We observed modest differences in basal and BZ-induced HSF1 mRNA levels between 253JB-V and UM-UC13 cells; specifically, 253JB-V showed a 2-fold increase in HSF1 levels upon drug exposure, whereas UM-UC13 showed only ∼1.3 fold increase, but had 2-fold higher HSF1 mRNA expression at baseline than did 253JB-V (Fig. 3A, left panel). However, protein levels appeared essentially equal between both cell types (Fig. 3A, right panel). Furthermore, other HSF1 targets were strongly induced, including the aforementioned HSPA1B (Fig. 2) and DNAJB1 (Hsp40) (Fig. S2) in the UM-UC10 and UM-UC13 cells suggesting that there was no generalized defect in endogenous HSF1 activation in these cells. We therefore reasoned that the UM-UC10 and UM-UC13 cells might possess specific defect(s) in HSF1-mediated activation of the HSPA1A promoter. Consistent with this idea, chromatin immunoprecipiation (ChIP) revealed that 253J B-V cells possessed higher levels of HSF1 binding to the HSPA1A promoter at baseline and following bortezomib exposure than did UM-UC13 (∼2-fold and ∼5-fold higher, respectively) (Fig. 3B). The fold-induction of HSF1 binding by bortezomib was ∼9-fold vs. ∼4-fold in 253JB-V and UM-UC13, respectively. Analysis of the HSPA1A promoter using the UCSC Genome Browser revealed that it lies within a CpG island that is methylated in other cancer cell lines (Fig. S3). Using methylation-specific PCR, we confirmed that the HSPA1A promoter was strongly methylated in the UM-UC10 and UM-UC13 but not in the 253J B-V or SW780 cells (Fig. 3C), which possibly accounted for defective bortezomib-induced HSPA1A induction. To directly test this possibility, we examined the effects of the histone methyltransferase inhibitor 5-aza-2′-deoxycytidine on basal and bortezomib-induced HSPA1A mRNA levels in the UM-UC10 and UM-UC13 cells. The inhibitor induced large increases in both basal and proteasome inhibitor-induced HSPA1A levels in both bortezomib-sensitive cell lines (Fig. 3D). Together, these results demonstrate that chromatin methylation is responsible for the defective HSPA1A induction observed in UM-UC10 and UM-UC13 cells.

### Modulation of HSPA1A and HSPA1B Expression in the HSPA1A^low^ Cells

**Figure 4:**
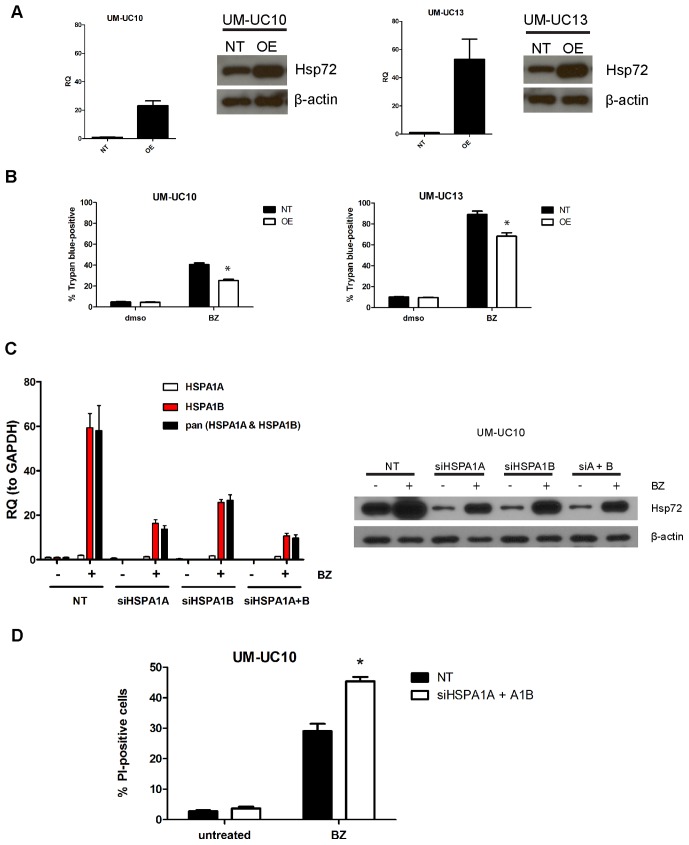
Genetic modulation of HSPA1A and HSPA1B in HSPA1A-low UM-UC10 and UM-UC13 cells. **A.** Effects of enforced HSPA1A overexpression on HSPA1A mRNA and Hsp72 total protein levels in UM-UC10 and UM-UC13 cells. Cells were stably transduced with a lentiviral HSPA1A expression construct, and HSPA1A levels were measured by quantitative RT-PCR. Mean ± SEM, n = 3. RQ = relative quantity (to GAPDH). Protein levels were measured via immunoblotting. Blots are representative of two independent experiments. **B.** Effects of HSPA1A overexpression on bortezomib-induced cell death. Cells transduced with empty vector (NT) or with the HSPA1A expression construct were exposed to 30 nM bortezomib for 24 h and plasma membrane integrity was measured by trypan blue uptake. (The presence of RFP in the expression construct prevented our use of the PI/FACS cell death assay.) Mean ± SEM, n = 3. *, P<0.02. **C.** Knockdown of HSPA1B in UM-UC10 cells. Left panel, knockdown efficiencies of siRNA against HSPA1A, HSPA1B, or both isoforms as measured by quantitative RT-PCR following exposure to 30 nM bortezomib for 6 h. RQ = relative quantity (to GAPDH). Right panel, corresponding knockdown of Hsp72 protein levels by the difference siRNA sequences following exposure to 30 nM BZ for 14 h. Data are representative of two independent experiments. **D.** Effect of HSPA1B knockdown on bortezomib-induced cell death in UM-UC10 cells. Following 72 h knockdown, cells were exposed to 30 nM bortezomib for 24 h and cell death measured by PI/FACS analysis. Values represent mean±SE (n = 3). *P<0.01.

Since UM-UC10 and UM-UC13 lacked HSPA1A expression, we examined whether replacing the HSPA1A isoform would promote bortezomib resistance. To address this, we stably overexpressed HSPA1A in both UM-UC10 and -UC13 cells using a lentiviral vector. HSPA1A mRNA expression was confirmed using qRT-PCR and Hsp72 total protein increases by immunoblotting (Fig. 4A). HSPA1A overexpressing cells and empty vector transduced cells were then exposed to bortezomib and we discovered that overexpression significantly reduced bortezomib-induced cell death (Fig. 4B). Conversely, because UM-UC10 and -UC13 cells appeared to be relying solely on HSPA1B mRNA for Hsp72 protein expression, we hypothesized that these cells may be particularly susceptible to targeting of HSPA1B. To test this, we used siRNA to transiently silence HSPA1B in UM-UC10 cells. Analysis of knockdown efficiencies revealed that the commercially available siRNAs cannot specifically target individual isoforms (i.e., siHSPA1A silenced HSPA1B) (Fig. 4C). Nonetheless, a combination of siHSPA1A and siHSPA1B sequences yielded the best (though not complete) overall knockdown of the A1B isoform at both the RNA and protein level (Fig. 4C). Using this strategy, we confirmed that blockade of HSPA1B induction sensitized UM-UC10 cells to bortezomib (Fig. 4D).

### Hsp72 Induction Inhibits Bortezomib-induced Cell Death

**Figure 5:**
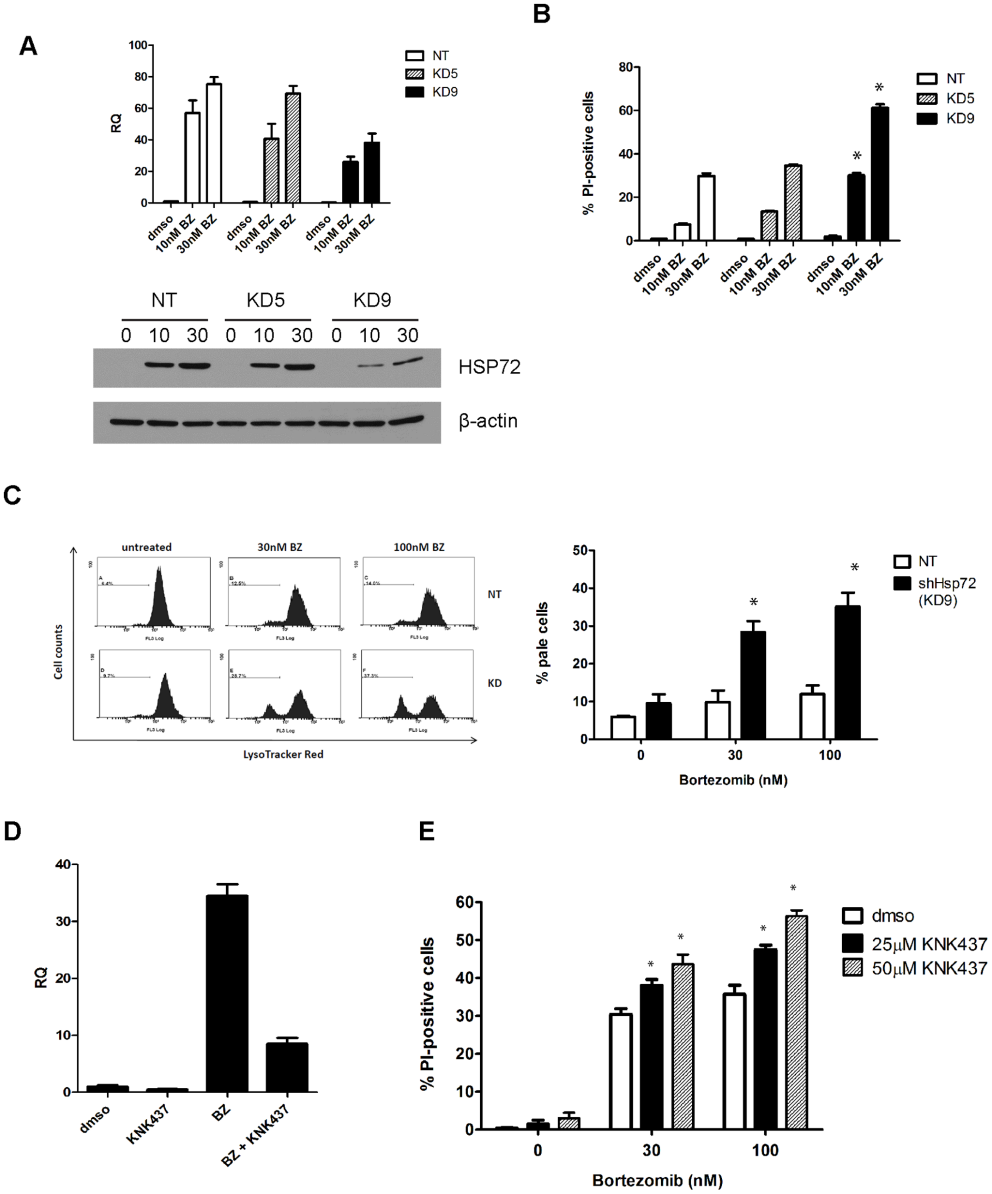
Inhibition of Hsp72 induction sensitizes resistant cells to bortezomib. **A.** Effects of stable Hsp72 knockdown on basal and bortezomib-induced Hsp72expression. 253J B-V cells were transduced with a non-targeting (NT) or HSPA1A/B (KD5, KD9) lentiviral shRNA constructs as described in Materials and Methods. Top panel, cells were incubated with or without indicated concentrations of bortezomib for 6 h and HSPA1A expression was measured by quantitative RT-PCR. Mean ± SEM, n = 3. RQ = relative quantity (to GAPDH). Bottom panel, effects of stable Hsp72 knockdown on Hsp72 protein levels. Cells were incubated for 12 h with indicated concentrations of bortezomib (nM), and Hsp72 levels were measured in whole cell lysates by immunoblotting. **B.** Effects of stable Hsp72 knockdown on bortezomib-induced cell death. Cells were incubated with the indicated concentrations of bortezomib for 48 h and PI/FACS analyses were used to quantify cell death. Mean ± SEM, n = 3. *, P<0.01 compared to corresponding NT values. **C.** Effects of stable Hsp72 knockdown on lysosomal membrane integrity. Cells were exposed to the indicated concentrations of bortezomib for 18 h prior to staining with Lysotracker Red, and loss of red fluorescence was measured by FACS. Left panel: representative FACS histograms. Right panel: results were quantified (mean±SEM; n = 3). *P<0.03. **D.** Effects of pharmacologic inhibition by KNK-437 on bortezomib-induced HSPA1A levels. Bortezomib-resistant 253J B-V cells were exposed to 25 µM KNK-437 with or without 100 nM bortezomib for 12 h, and HSPA1A levels were measured by quantitative RT-PCR. Mean ± SEM, n = 3. RQ = relative quantity (to GAPDH). **E.** Effects of KNK-437 on cell death. 253J B-V cells were exposed to 25 or 50 µM KNK-437 in combination with 30 or 100 nM bortezomib for 48 h, and loss of plasma membrane integrity was quantified by PI/FACS. Mean ± SEM, n = 3. *, P<0.05.

To more directly determine whether bortezomib-induced Hsp72 upregulation promoted resistance, we stably knocked down Hsp72 in 253JB-V bortezomib-resistant cells using a lentiviral shRNA vector (Fig. 5A). Baseline HSPA1A mRNA levels were reduced by more than 75% in the cells, but shRNA-mediated suppression of HSPA1A mRNA (Fig. 5A. upper panel) and Hsp72 protein (Fig. 5A, lower panel) was less impressive following exposure to bortezomib, presumably because the proteasome inhibitor produced such a strong upregulation of Hsp72. Nonetheless, stable Hsp72 knockdown significantly enhanced bortezomib-induced loss of plasma membrane integrity as measured by propidium iodide uptake (Fig. 5B). Previous studies concluded that Hsp72 induction serves a cytoprotective function within the integrated stress response (ISR) by stabilizing lysosomes [29]. As such, we compared the effects of bortezomib on lysosomal integrity in the 253JB-V cells transduced with control vector (253JB-V NT) or the KD9 HSPA1A-specific shRNA construct. Bortezomib had little to no effect on lysosomal integrity in the 253JB-V NT cells but induced strong, concentration-dependent loss of lysosomal integrity in the 253JB-V-KD9 cells (Figure 5C). Together, these results confirm that bortezomib-induced Hsp72 induction functions to promote lysosomal integrity and to inhibit cell death. Finally, we examined whether pharmacologic HSF1 inhibition would also promote bortezomib-induced cell death. The chemical HSF1 inhibitor KNK-437 strongly attenuated bortezomib-induced HSPA1A induction (Fig. 5D) and promoted cell death (Fig. 5E) in the 253JB-V cells. These data support the idea that chemical inhibitors of HSF1 and/or Hsp72 can be used to promote bortezomib-induced cell death.

### Hsp72 Knockdown Promotes Bortezomib-induced Tumor Growth Inhibition in vivo

**Figure 6:**
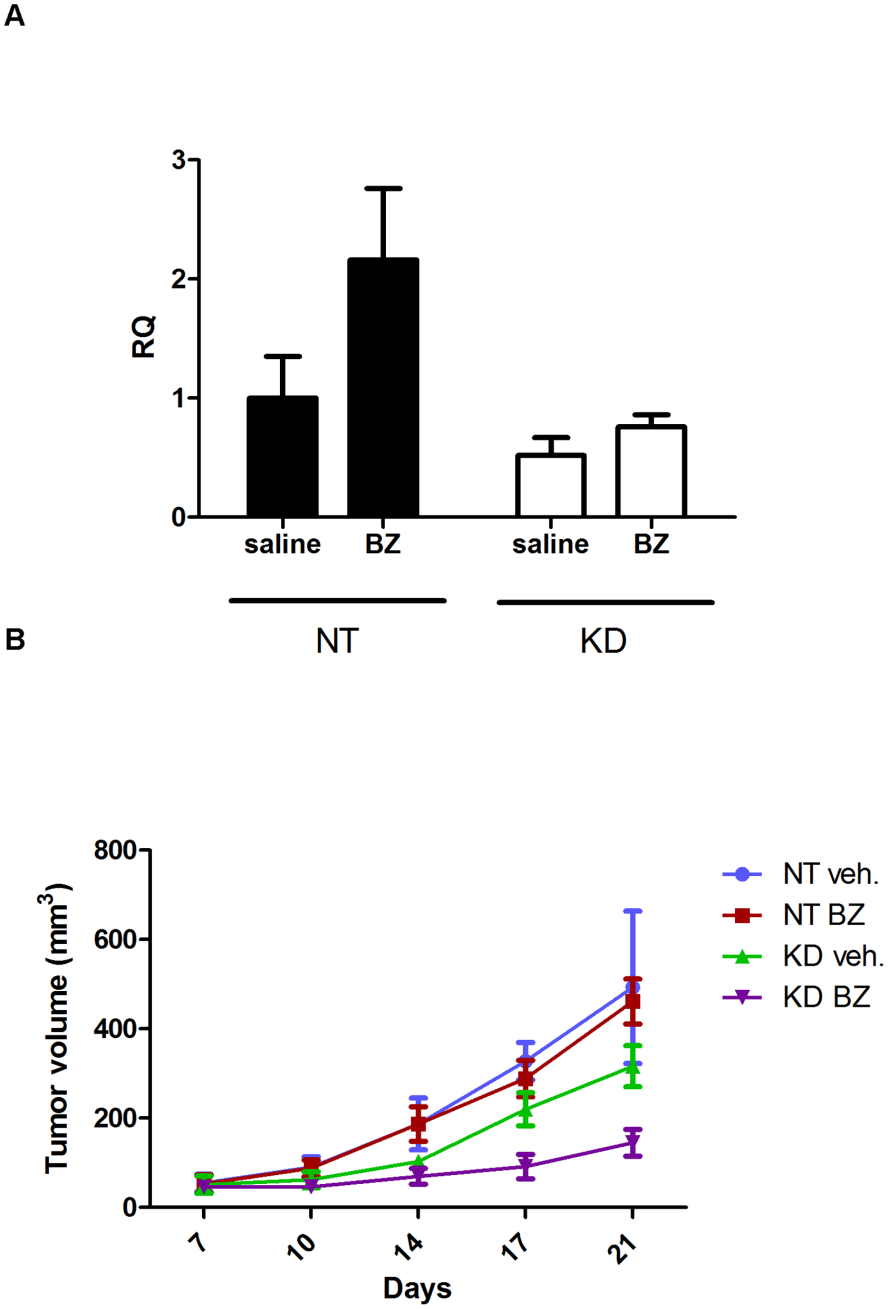
Knockdown of HSPA1A promotes bortezomib-induced growth inhibition *in vivo*. **A.** Effects of bortezomib on HSPA1A induction. Animals were injected twice with 1 mg/kg bortezomib (3 days apart), tumor RNA was harvested, and HSPA1A expression was measured by quantitative RT-PCR. Mean ± SEM, n = 3. RQ = relative quantity (to GAPDH). **B.** Effects of bortezomib on tumor growth. Athymic nude mice were inoculated s.c. with 253JB-V.KD9 or 253JB-V.NT cancer cells. When tumors became palpable (5–7 days), the mice were treated i.v. biweekly with bortezomib at 1 mg/kg/dose or with saline control. Tumor volumes were measured twice a week after the start of treatment. Values represent mean ± SE (N = 5).

In a final series of experiments we examined whether stable Hsp72 knockdown would promote the growth-inhibitory effects of bortezomib in 253JB-V tumors in vivo. We established subcutaneous tumors using 253J B-V cells transduced with either the non-targeting (NT) or Hsp72-specific KD9 shRNA constructs and dosed animals with bortezomib (1 mg/kg, the MTD) twice weekly via i.v. injection. Using quantitative real-time RT-PCR, we confirmed that bortezomib increased HSPA1A mRNA levels in vivo and that the shRNA construct inhibited these effects (Fig. 6A). The untreated 253JB-V KD9 tumors displayed somewhat slower tumor growth than did the 253JB-V NT tumors, but the differences did not reach statistical significance. Biweekly therapy with bortezomib had no significant effects on the growth of the control 253JB-V.NT tumors (Fig. 6B), consistent with our previous findings [17]. Conversely, bortezomib almost completely suppressed the growth of the tumors derived from the 253JB-V cells transduced with the HSPA1A-specific shRNA construct (Fig. 6B).

## Discussion

In this study we demonstrate for the first time that the major inducible chaperone within the Hsp70 family, Hsp72, promotes resistance to bortezomib in bladder cancer cell lines. Induction of Hsp72 protected the resistant bladder cancer cells from the cytotoxic effects of bortezomib *in vitro* and *in vivo*. Hsp72 plays a well-established role in the ISR by preventing peptide nascent-chain misfolding and subsequent aggregation [30], by stabilizing lysosomes [31], and by directly binding to and inhibiting pro-apoptotic factors such as Apaf-1, AIF, and JNK [32]. A strong body of literature highlighting the role of Hsp72 in lysosomal function/integrity [29], [33] prompted us to investigate and positively identify increased levels of lysosomal instability as a mechanism for the increased bortezomib sensitivity displayed in the Hsp72-silenced 253JB-V cells. These results coincide with evidence citing the autophagy-lysosomal system as a critical regulator of cellular response to proteasome inhibitors [34]. Indeed, we previously demonstrated that modulating the lysosome-dependent process of autophagy can sensitize cancer cells to bortezomib [3]. We speculate that Hsp72-mediated lysosomal stabilization is required for efficient autophagic clearance of bortezomib-induced protein aggregates, and that inhibiting these effects promotes cytotoxicity.

The differential expression of HSPA1A in the four bladder cancer cell lines tested was associated with differential binding of HSF1 to the HSPA1A promoter, which was due to HSPA1A promoter methylation in the UM-UC10 and UM-UC13 cells. The histone methyltransferase inhibitor 5-aza-2′-deoxycitidine restored both baseline and bortezomib-induced HSPA1A expression, confirming that HSPA1A promoter methylation underlies the defect in gene induction observed in the UM-UC10 and –UC13 cells. In preliminary experiments we have determined that HSPA1A is also methylated in approximately half of primary human bladder cancers (W. Qi, unpublished observations). HSPA1A was also recently found to be hypermethylated in ovarian cancer cells [35]. At present, we do not have an explanation for why a major isoform of such an important chaperone would be silenced in a large fraction of human bladder cancers. However, an attractive explanation is that the HSPA1A gene lies in a particularly vulnerable CpG island that is targeted incidentally as a result of the more global methylation changes that drive bladder cancer cancer progression [36]. HSPA1A is located on the 6p21.3 region of chromosome 6 within a gene cluster best known as the MHC region (major histocompatibility complex), noted for its high density of MHC class I-III genes. HSPA1B is also located in this same region, but is thought to have a different promoter [37], providing the cell with two different loci from which to obtain Hsp72 protein should one promoter be turned off. The importance of maintaining Hsp72 protein levels is highlighted by our discovery of dramatic compensatory upregulation of the HSPA1B isoform in bladder cancer cells that lack the A1A isoform. In spite of the promising correlation between HSPA1A methylation and bortezomib sensitivity in the 4 cell lines characterized here, in other preliminary experiments using bladder cancer cell lines, we have found that HSPA1A expression does not appear to correlate well with bortezomib sensitivity (M. White, unpublished results). However, other functional consequences of the epigenetic silencing of HSPA1A in cancer should be explored with regard to tumor biology and chemotherapeutic response. More importantly, methylation of HSPA1A renders cells completely dependent upon HSPA1B for Hsp72 expression. Developing strategies to specifically inhibit HSPA1B could produce synthetic lethality in bladder cancers and other tumors with HSPA1A methylation.

There is growing enthusiasm in multiple disease sites for therapeutic modulation of the proteostasis network. Cancer cells in particular display higher levels of molecular chaperones [38] and pirate the protective functions of HSF1 to support their transformation [39], [40]. The development of Hsp90 inhibitors has firmly established protein chaperones as valid clinical targets, and agents such as the geldanamycin analogue 17-AAG, IPI-504 (retaspimycin), and VER52296 are currently in clinical trials for cancer [41]. In contrast, the availability of Hsp72 and HSF1 inhibitors is noticeably lacking. HSF1 inhibitors including triptolide, KNK-437, quercetin, NZ28, and emunin are limited by poor specificity and potency [42]. 2-phenylethynesulfonamide (PES) was recently shown to be a relatively specific chemical inhibitor of inducible Hsp72, disrupting multiple Hsp72 functions while avoiding interaction with other chaperones such as Hsc70, Grp78, or Hsp90 [43]. A small number of other Hsp72 inhibitors have been reported in preclinical studies, including ADD70 (AIF-derived decoy for Hsp70) [44], VER-155008 [45], and the dihydropyrimidine MAL3 compounds [46]. Collectively, our results support the further evaluation of combination therapy with bortezomib plus Hsp72 and/or HSF1 inhibitors in xenograft models of bladder cancer to determine toxicity and therapeutic efficacy and encourage the continued development of more potent heat shock response inhibitors.

## Supporting Information

Figure S1 **Whole-genome expression profiling depicting effects of bortezomib on HSR gene expression in 253JB-V and UM-UC13 cells.** Cells were incubated with or without bortezomib for 6 or 12 h, and global gene expression patterns were compared using the Illumina platform. Arrows highlight HSPA1A and HSPA1B.
(TIF)

Figure S2 **Bortezomib-induced expression of other heat shock proteins.** Cells were exposed to 30nM BZ for 6 h, and mRNA expression changes were measured via quantitative RT-PCR. Values represent mean±SE (n = 2). Top, DNAJB1 (Hsp40); middle, HSPA8 (Hsc70); bottom, HSPB1 (Hsp27).
(TIF)

Figure S3 **The HSPA1A promoter contains a CpG island that is commonly methylated in cancer.** **A.** HSPA1A gene locus on Chromosome 6. Specific location is 6p21.3. **B.** UCSC Genome Browser screenshot depicting a CpG island (dark green bar) at HSPA1A consensus promoter regions in multiple cell lines (red bars). Below, DNA methylation analysis of the HSPA1A promoter region across multiple cell lines and tissues types. Unmethylated = green; 50% methylated = yellow; 100% methylated = red. Note that 8 out of 9 cell lines with significant methylation (orange-red color) were derived from human tumors.
(TIF)
