# Supplementary figures and images for: Inhibition of Inducible Heat Shock Protein-70 (Hsp72) Enhances Bortezomib-Induced Cell Death in Human Bladder Cancer Cells
Source: PLoS One. 2013 Jul 18;8(7):e69509. doi: 10.1371/journal.pone.0069509 (PMC3715471; doi:10.1371/journal.pone.0069509)

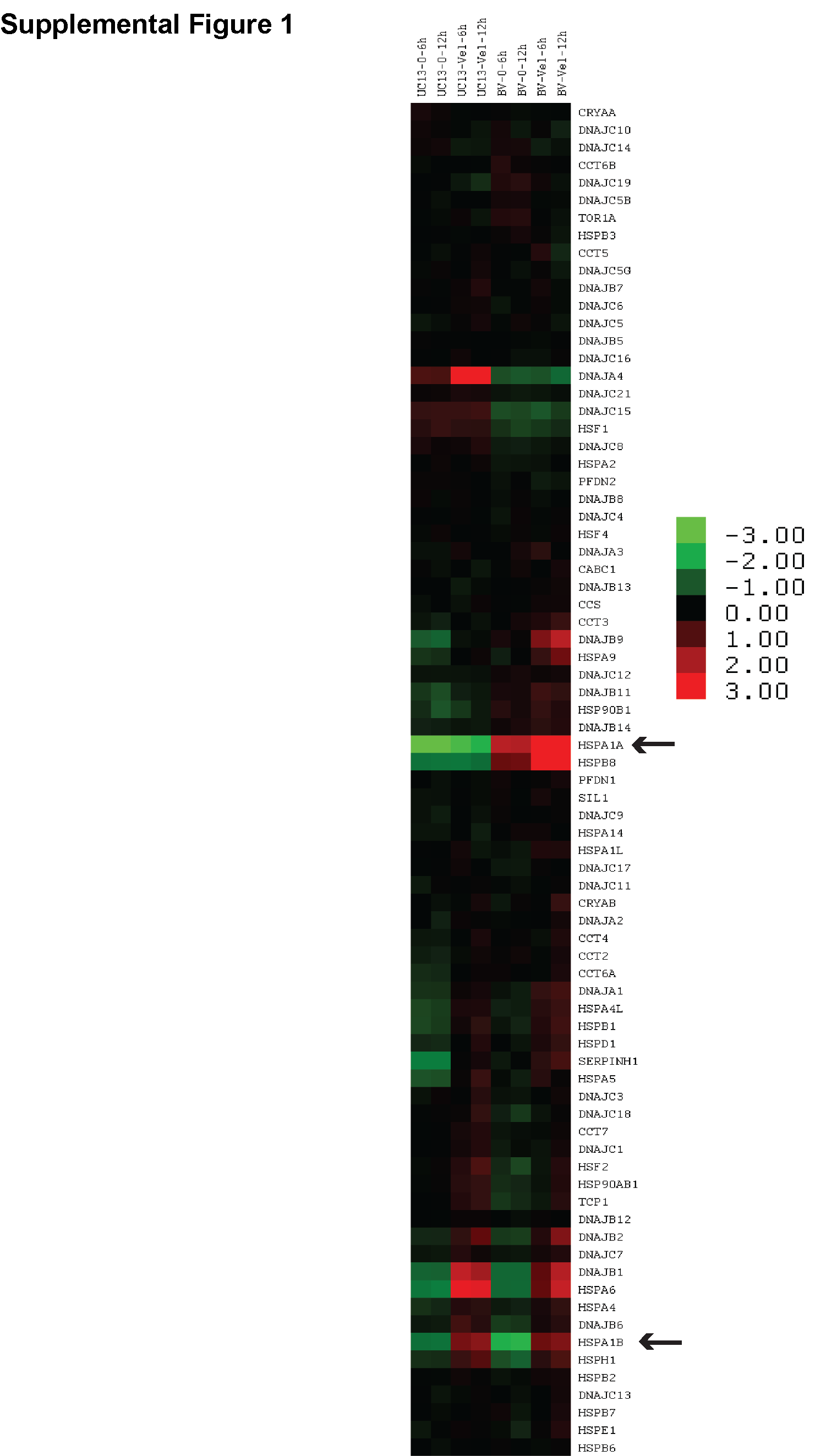

Supplement: Figure S1 — Whole-genome expression profiling depicting effects of bortezomib on HSR gene expression in 253JB-V and UM-UC13 cells. Cells were incubated with or without bortezomib for 6 or 12 h, and global gene expression patterns were compared using the Illumina platform. Arrows highlight HSPA1A and HSPA1B. (TIF) [file pone.0069509.s001.tif]

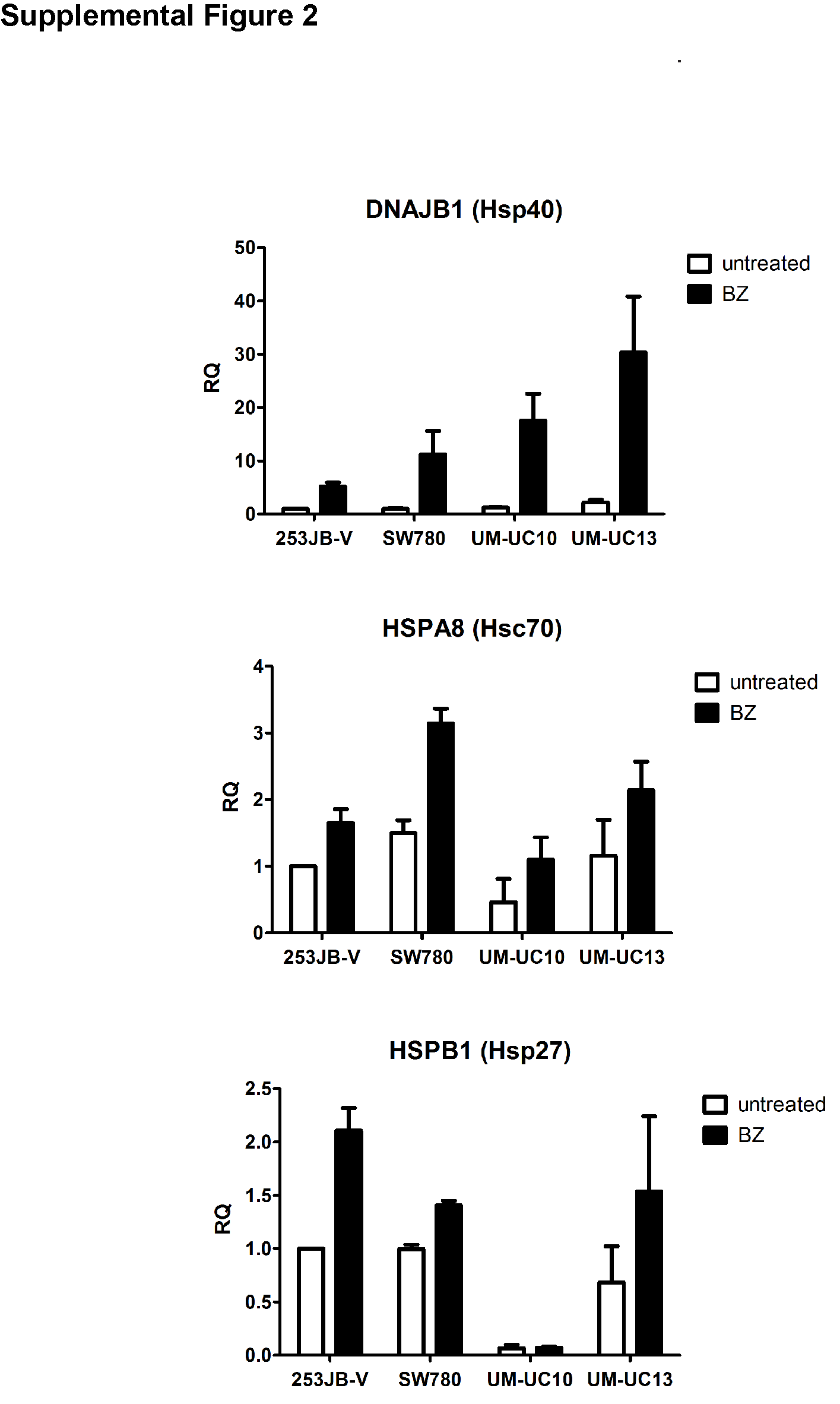

Supplement: Figure S2 — Bortezomib-induced expression of other heat shock proteins. Cells were exposed to 30nM BZ for 6 h, and mRNA expression changes were measured via quantitative RT-PCR. Values represent mean±SE (n = 2). Top, DNAJB1 (Hsp40); middle, HSPA8 (Hsc70); bottom, HSPB1 (Hsp27). (TIF) [file pone.0069509.s002.tif]

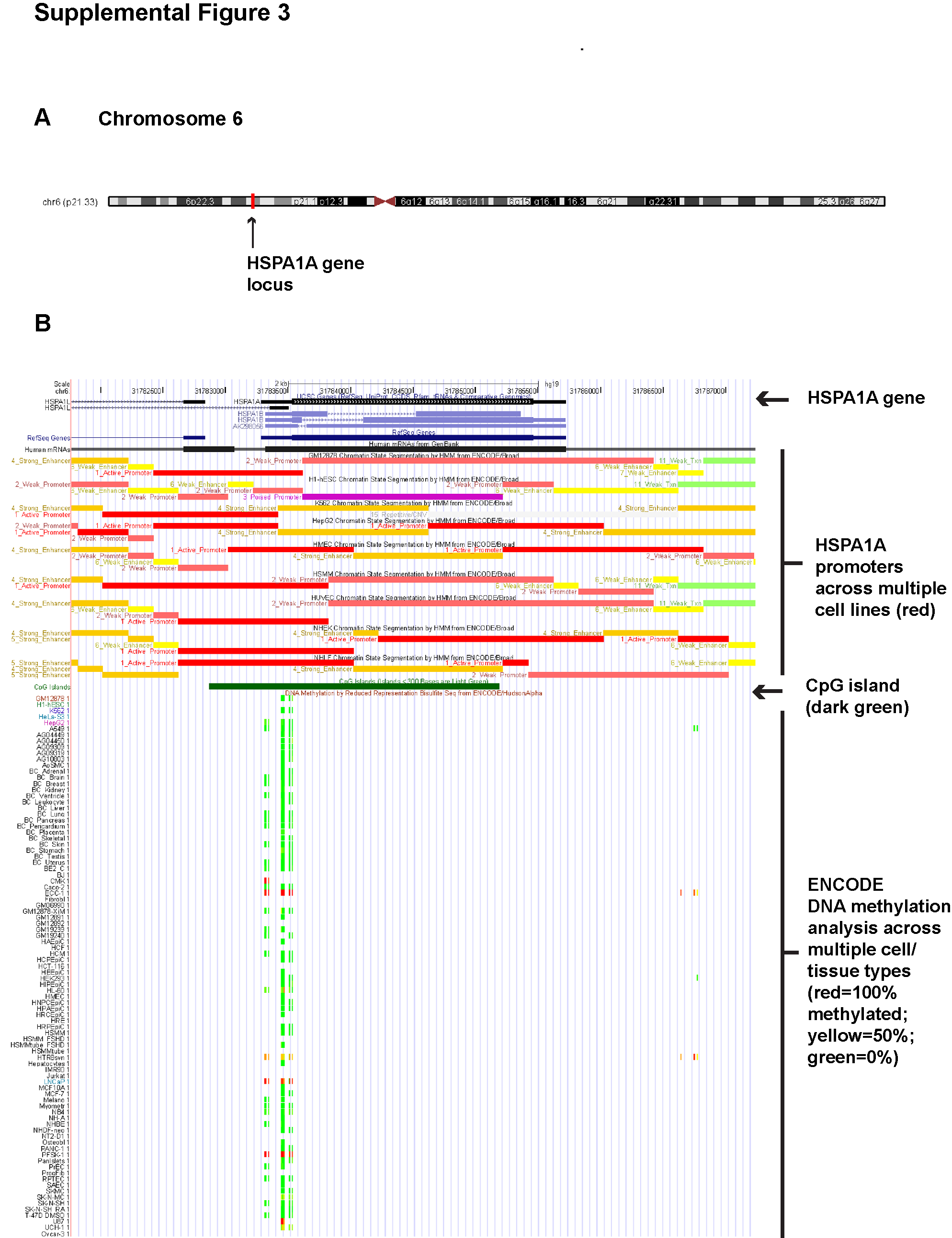

Supplement: Figure S3 — The HSPA1A promoter contains a CpG island that is commonly methylated in cancer. A. HSPA1A gene locus on Chromosome 6. Specific location is 6p21.3. B. UCSC Genome Browser screenshot depicting a CpG island (dark green bar) at HSPA1A consensus promoter regions in multiple cell lines (red bars). Below, DNA methylation analysis of the HSPA1A promoter region across multiple cell lines and tissues types. Unmethylated = green; 50% methylated = yellow; 100% methylated = red. Note that 8 out of 9 cell lines with significant methylation (orange-red color) were derived from human tumors. (TIF) [file pone.0069509.s003.tif]
